# Implementation and performance barriers in Iran’s breast cancer screening program: a qualitative case study

**DOI:** 10.3389/fpubh.2025.1490191

**Published:** 2025-05-09

**Authors:** Arezoo Jabbari, Zhila Najafpour, Sima Ourang, Somayeh Loveimi, Reyhane Bohrani, Mahsa Baymani

**Affiliations:** ^1^Department of Health Care Management, School of Public Health, Ahvaz Jundishapur University of Medical Sciences, Ahvaz, Iran; ^2^Department of Health Care Management, School of Public Health, Cancer Research Center, Ahvaz Jundishapur University of Medical Sciences, Ahvaz, Iran; ^3^Department of Non-Communicable Diseases, Deputy of Health, Ahvaz Jundishapur University of Medical Sciences, Ahvaz, Iran

**Keywords:** barriers, health system and services, cancer screening program, breast cancer, qualitative

## Abstract

**Introduction:**

Cancer screening programs (CSPs) are essential for early detection and improving survival rates; however, they often encounter barriers to effective implementation. This study aims to identify the key challenges faced in implementing the Breast Cancer Screening Program (BCSP) in Iran, with the goal of providing insights to enhance the program’s effectiveness and accessibility.

**Methods:**

This study was conducted as an observational and qualitative research to assess the implementation status and identify barriers within the Breast Cancer Screening Program (BCSP). Data were collected through a combination of interviews, process observations, and document reviews. A purposive sample of 37 participants was interviewed, including individuals involved in the management, implementation, or evaluation of the screening program. Thematic content analysis was employed to analyze the data, with saturation achieved to ensure comprehensive coverage of the study’s objectives.

**Results:**

The study identified several key barriers to the effective implementation of breast cancer screening programs, which were categorized into three main dimensions: infrastructural, managerial, and healthcare service delivery. The most critical issues in infrastructural category were a lack of trained healthcare personnel, insufficient screening facilities, inaccurate registration systems, fragmented databases, and poor data quality control. Key barriers in the managerial dimension include the absence of a mechanism for identifying and inviting women eligible for cancer screening, as well as inadequate monitoring of non-responders to follow-up. Overcrowding during peak times, long waiting periods, inaccurate triage, and lack of general practitioners (GPs) at the primary level of public healthcare were the identified barriers in service delivery in cancer screening. Additionally, interviews with women revealed several barriers, such as low perceived risk, fear and anxiety, lack of family support, and cultural or religious objections, all of which further hindered participation in breast cancer screening.

**Conclusion:**

This study highlights the critical barriers to the implementation of breast cancer screening programs in Iran, most of which appear to stem from systemic failures. Addressing these challenges requires a comprehensive, strategic approach that targets the identified obstacles at multiple levels. Overcoming these barriers is crucial to improving the accessibility, efficiency, and overall effectiveness of breast cancer screening programs, ultimately enhancing early detection and patient outcomes.

## Introduction

Among women, breast cancer accounted for approximately 24.5% of all cancer cases and 15.5% of cancer deaths, ranking first for incidence and mortality in the majority of the world countries in 2020. Breast cancer is one of the most prevalent types of cancer in Iran and are expected to remain the leading cancer nationally in 2025 (1). Estimates in Iran show from 2016, the trend of breast cancer has been increasing and this growth is expected to continue until 2040 when the number of cancers will more than double compared to 2020 (1). Based on a literature, the prevalence of breast cancer in Iranian women was 34.5 per 100,000 (2, 3).

Based on the evidence, early detection of cancer plays a pivotal role in future therapeutic methods and significantly improves the chances of successful treatment (4). Romero et al., reported that that there were fewer breast cancer early detection programs in Low and Middle Income Countries (LMICs) compared with high-income countries (5). Then, late-stage breast cancer diagnoses are more common in low- and middle-income countries (LMICs). Evidence shows that in high-income countries (HICs), 70% of breast cancer cases are diagnosed at stages I or II, whereas in LMICs, fewer than 50% of patients are diagnosed at these early stages (6).

Screening is an essential step in the early diagnosis of diseases that can detect breast cancer before it has any symptoms (7). Population-based screening programs can be personalized, categorizing women into different groups based on individual risk factors and preferences (8). One of the goals of implementing organized breast cancer screening programs is to achieve high participation rates among the target population and maintain consistent follow-up in breast cancer screening (9). Like most of developed and developing countries (10, 11), Iran’s National Cancer Control program (IRCCP) was developed comprehensively in 2013 with cross-sectoral cooperation and stakeholder participation. Early detection of breast cancer is one of the targets within this program (12).

Despite substantial advances in the early diagnosis and treatment of breast cancer, it remains one of the leading causes of cancer-related mortality among women (13). Several methods are recommended for breast cancer screening, including digital mammography, digital breast tomosynthesis, breast ultrasonography, magnetic resonance imaging (MRI), and clinical breast examination (CBE) (14). Currently, mammography is regarded as an effective screening method for detecting breast cancer at its early stages (15). Based on the results of a study in Iran, more than half of the participants underwent breast self-examination (BSE), clinical breast examination (CBE), and mammography at least once. However, only a small percentage performed these examinations regularly and in accordance with recommended guidelines. Specifically, 9.9% conducted BSE regularly once per month, 8.9% underwent CBE regularly twice per year, 12.3% had mammography regularly once per year, and 3.8% received sonography regularly twice per year (16). Another study is reported the BSE, CBE and mammography rates were 4.1, 5.6 and 4.8%, respectively (17). These rates fall short of the established targets when compared to those in other countries (18, 19).

Based on literature, well organized cancer plans that are linked to strong governance mechanisms improve cancer outcomes. However, many of these plans are not being implemented because of underfunding, inadequate expertise for scale-up, competing priorities, or lack of political will. Based on the WHO report from 2015, about one in four countries do not operationalize their National Cancer Control Plan (NCCP) or Non-Communicable Disease (NCD) plan (5, 10). Therefore, in light the importance of implementing a breast cancer screening program for early detection and timely treatment, it is crucial to evaluate the program to identify any underlying challenges. This research aims to identify systemic obstacles by evaluating breast cancer screening through interviews with both healthcare providers and women.

## Materials and methods

This research was carried out in two phases: an observational study to extract the workflow of the breast cancer screening program, and a qualitative study to identify the challenges of the program from the perspectives of both health service providers and women.

### Extraction of the breast cancer screening workflow

To ensure a comprehensive understanding and enhance the reliability and validity of the findings, we employed a triangulation approach by integrating three data collection methods: interviews, direct observations, and document analysis.

- Interviews: 11 healthcare providers directly involved in the screening process were interviewed. The purpose of these interviews was to gain detailed insights into the routine workflow, including the sequence of tasks, roles of different staff members, and standard procedures followed during screening. The interviews helped to map out the step-by-step process and identify any variations across different healthcare centers. The participants included midwives (*n* = 2), healthcare workers (*n* = 3), physicians at health service centers (*n* = 2), executive directors of health service centers (*n* = 2), and staff from the Deputy of public health, including the heads of the non-Communicable diseases group (*n* = 1) and the family health group (*n* = 1).
- Process Observation: The researchers (A.J., S.L.) observed the workflow at four Comprehensive Health Service (CHS) centers and one Early Detection Cancer (EDC) center. These observations focused on key operational processes, including patient admission, patient flow, staff interactions, information registration in databases, screening procedures, and the use of medical records, diagnostic tools, and patient discharge. The researcher systematically documented the steps involved in the cancer screening program. Observations were conducted during regular working hours over multiple days to capture routine practices and potential challenges in implementing screening protocols. After identifying the various components of the workflow and reaching saturation of results without discovering new steps, the researcher developed a framework for implementing the screening program.
- Document analysis: A comprehensive review of the relevant documents was also undertaken, including national guidelines related to breast cancer screening, to assess their alignment with current practices in the field (See details in Supplementary 1).

### Qualitative phase

Following the extraction of the workflow of the BCS program, the researchers evaluated the process for potential challenges through face-to-face interviews.

The study sample is categorized in two parts health care providers and women. In this study, purposeful sampling was employed to select health providers with specific knowledge, or expertise relevant to screening programs.

- In the first group, we interviewed with involved staff in providing breast cancer screening services including midwives, nurses, physicians, executive directors, and managers from the public health deputy (such as the director of the non-communicable diseases department, the family health group, and staff responsible for the registration and monitoring of the program’s implementation and referrals for screening and breast cancer patients). The inclusion criteria for health providers specified that individuals must have at least 3 years of executive or supervisory experience in breast cancer screening.
- In the second group, we used convenience sampling method. The inclusion criteria for the participants were as follows: women aged 30 years and older, with no previous or current diagnosis of breast cancer, and women who did participate during the screening process at the first or the second-level health centers.

Interviews were conducted until data saturation was achieved. Saturation was reached after we thoroughly explored all the interview questions, and no new themes emerged during subsequent interviews with participants, resulting in a total of 37 participants (14 health providers and 23 women). It is noteworthy to mention, 11 participants from Phase 1, who were interviewed to identify the steps of breast cancer screening program, also participated in phase 2, where the challenges in the program were explored.

The interview questions were designed as open-ended and based on a thorough review of relevant studies. To effectively capture the challenges associated with implementing the breast cancer screening program, the questions were divided into two categories: existing challenges from the perspectives of healthcare professionals and women.

- Healthcare professionals’ perspective: The questions addressed the perspectives of the healthcare providers across six domains—human resources, information management, technology, governance and leadership, financial aspects, and drugs and equipment—and included a total of 20 questions (See details in Supplementary 2).
- Women perspective: We explored women’s knowledge and attitudes due to seven questions toward breast cancer risk factors, their previous experiences with mammography and clinical breast examinations, and recommendations for mammography frequency across various age groups and risk level. We also examined their concerns about current screening practices and the factors influencing their decisions to either undergo or forgo mammograms and clinical breast examinations. Additionally, we investigated the role of social support and communication with healthcare professionals and family, as well as barriers and facilitators related to access, cost, and physical issues within the screening program system (See details in Supplementary 2).

### Conduct interviews and data collection

Each interview lasted approximately of 30–35 min. At the beginning of each interview, the purpose of the study was explained, consent to record the interview was obtained, and participants were assured of the confidentiality of the content, including the anonymization of any examples used. Data collection continued until 70% saturation was reached, meaning no new information was emerging from subsequent interviews. Following the interviews, key components relevant to the study objectives were identified and categorized. Two researchers (Z.N. and A.J.) then organized these components into themes, categories, and subcategories. The classifications were compared, and any discrepancies were resolved through discussion. In cases of disagreement, a third party’s opinion was sought, leading to a final consensus. Participant quotations corresponding to each main theme were subsequently compiled and presented for clarity and reference.

### Data validation

Four criteria including credibility, confirmability, transferability, and dependability were used to maintain the trustworthiness of the extracted themes. Credibility was boosted through prolonged engagement with interviewees, the achievement of data saturation, and the sampling method. Also, member check supported credibility. After data analysis, participants were provided a complete transcript of their coded interviews. They confirmed the extracted themes. Meanwhile, maximum variant sampling (different positions, backgrounds) also validated the confirmability of data. In the case of reliability of study results, we asked other researchers in the field to assess the coding process. They were two experts who were experienced in qualitative research. They checked the transcripts of interviews and coded them as well. Then reliability of the coding was calculated to the number of agreed codes over the total number of codes for Inter-Rater Reliability (IRR). A score of more than 70% is considered a desired agreement. Additionally, the results were discussed with two people—experts in the field of non-communicable disease- who did not participate in our interview but who confirmed the soundness, fitness and transferability of the results. This confirmed transferability of results.

### Data analysis

Thematic content analysis was employed to analyze the data obtained from interviews, which were presented in textual form. The primary tool used in content analysis is the categorization system, in which each textual unit is coded and assigned to one or more categories. Each category was defined to be clear, comprehensive, and relevant, with the aim of capturing the text’s meanings as fully as possible while avoiding generalization and ensuring completeness. The analysis of the interviews utilized a comprehensive (inductive) approach, focusing on the criteria of the screening program. Two members of the research team independently reviewed the interviews. The results were found to be over 90% consistent.

## Results

A total of 37 participants took part in the study. To identify the steps involved in breast cancer screening, 11 healthcare providers were interviewed. In order to explore challenges in the breast cancer screening program, 14 healthcare providers and 23 women were interviewed. The characteristics of the interviewees are detailed in Table 1. Most interviewees were aged between 40 and 49 years (40.5%). Among the women, 39% had a primary education level, while 27% of healthcare professionals held a bachelor’s degree, and 50% of them had over 10 years of work experience.

**Table 1:** Participants characteristics (*n* = 37).

	Health care providers	Women
Age, years, mean ± SD	44.28 (10.43)	44.13 (6.33)
Education level		
No-formal education	0	6
Elementary school	0	3
Middle and high School	0	2
Diploma	0	10
College level	14	2
Work History		
5<	3	22
5-10	4	1
>10	7	0

### Breast cancer screening steps in Iran

The process of breast cancer screening (BCS) program is extracted from interviews within the primary and secondary levels of the health care system in Iran, begins with women attending a comprehensive health care center (CHCC) for a clinical breast examination. It is important to note that the target population is not actively recruited into the BCS program. Then, participants were women who were either highly motivated to engage in screening or who sought other healthcare services at the CHCC. Following this, midwives assessed these women using a structured questionnaire to determine their risk levels and subsequently conducted physical examinations to identify any abnormal breast masses.

During this process, women are educated on breast self-examination (BSE) for at-home use. For women with normal breast tissue aged between 30 and 40, a follow-up period of 2 years is recommended, whereas women older than 40 are advised to have follow-ups annually. Midwives refer patients who are identified as high-risk based on their medical history or those with abnormal clinical breast examinations to an EDC center at the secondary care level.

**Figure 1:**
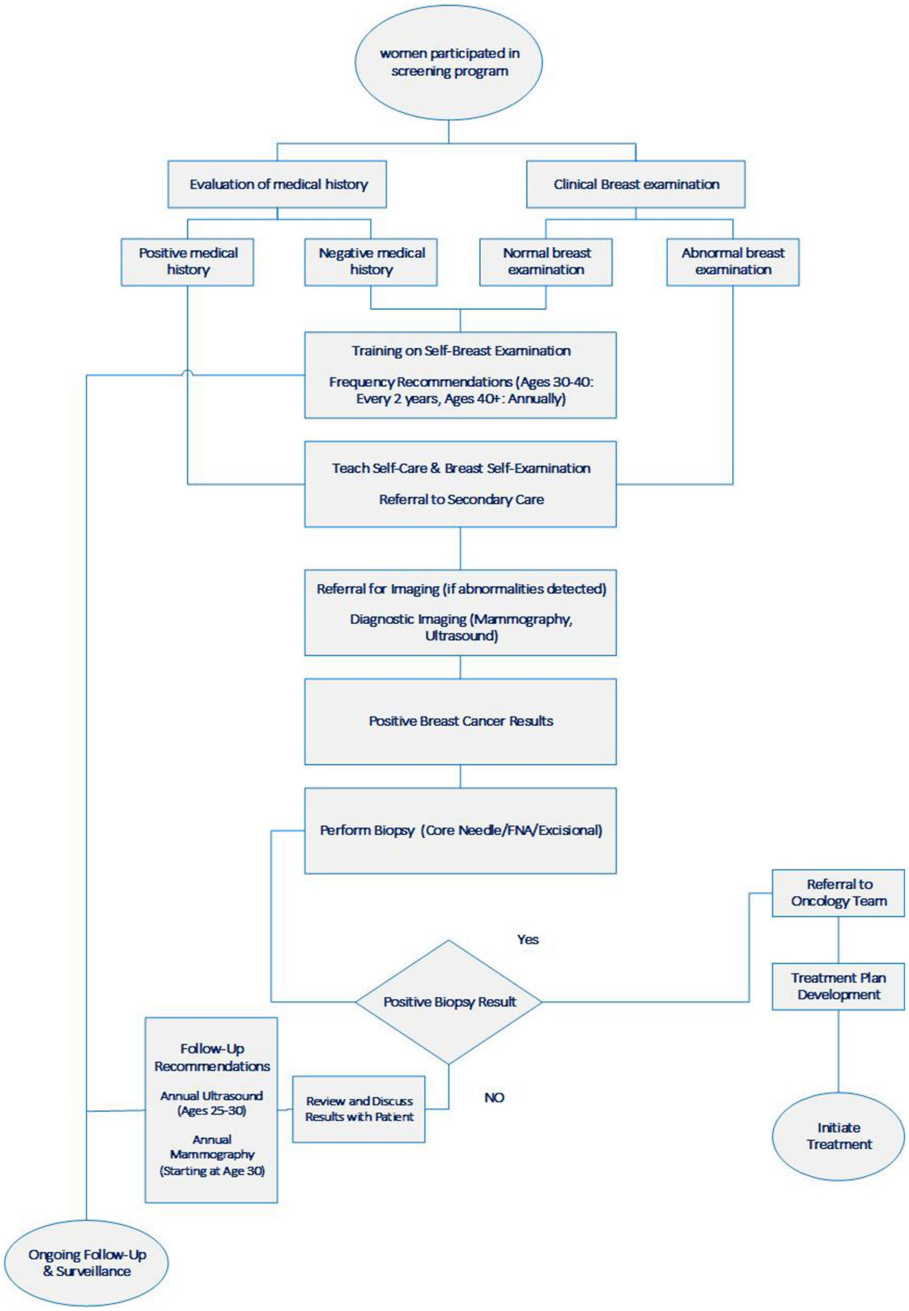
Breast cancer screening steps in Iran.

Following a positive result from breast screening sonography or mammograms indicating malignancy, patients are referred for breast tissue sampling (needle biopsy) or additional imaging to determine the stage and grade of the cancer. Meanwhile, for women with negative mammograms, annual sonography is recommended for those aged between 25 and 30, and annual mammography is recommended for those aged 30 and older (see Figure 1).

### Challenges in the breast cancer screening program

The identified challenges in the breast cancer screening program are categorized into three main dimensions: (a) infrastructural barriers, (b) managerial-related barriers, and (c) healthcare service delivery barriers (Refer to Conceptual Framework in Supplementary 3).

(a) Infrastructural Barriers: The findings indicate that the infrastructural dimension encompasses four key themes: human resources for health (HRH), physical infrastructure, health information systems, and medical facilities.

The main categories in the HRH were related to the quantity and quality of personnel. Identified barriers include restricted access to screening due to a shortage of skilled providers knowledgeable about screening guidelines and the high workload of primary care providers (such as midwives and general practitioners). Additional challenges include an insufficient number of specialist physicians at the secondary care level and a lack of motivation among healthcare providers due to inadequate compensation (see Table 2).

The findings reveal several concerns related to physical infrastructure, including the presence of only one EDC center at the secondary care level, the absence of changing rooms in primary care centers, and the lack of dedicated screening rooms for breast examinations. Additionally, there is a shortage of EDC centers in other cities and transportation barriers to accessing the early detection cancer center due to geographic location (see Table 2).

**Table 2:** Infrastructural barriers.

Theme	Category	Subcategory	Quotes
Human Resources for Health (HRH)	Quantity	Insufficient number of specialist physicians at the secondary care level	“Many of centers do not have doctors, especially in our urban areas.” “The early detection cancer center depends a lot on specialist doctors. If one decides to leave, we cannot find a replacement like her.”
		Lack of general practitioners in comprehensive health service centers in the primary care level	
		Inadequate number of skilled providers knowledgeable about screening guidelines	
		Shortage of midwives and healthcare workers in comprehensive health service centers in the primary care level	
	Quality	Lack of motivation among healthcare providers due to insufficient payment	“It does not make sense to have specialists at the early diagnosis center since I do not get paid the same way other specialists in hospitals.”
		Instability in the workforce and the use of the temporary staff	
		Weaknesses in continuing education programs	
		Screening tasks are often deprioritized due to the high workload of healthcare workers	
Physical Infrastructure	Facilities	Lack of suitable screening rooms for breast examinations	“I do not think this location for breast exams is ideal. We do not have a proper room to ask about medical history, educate women, or do the breast assessments.
		Limited number of early cancer detection centers in the province (only one center)	
		Inappropriate locations of comprehensive centers in cities	
		Weaknesses in welfare facilities at comprehensive health and early detection cancer centers	
Health Information System	Databases Linkage	Lack of linkage between SIB and other databases (governmental, private, and cancer registry)	“The SIB database is pretty good and has everything we need, but the issue is it’s not connected to the main screening center in the province. So, we cannot really follow up on suspicious cases effectively.”
		Absence of electronic health records at the early detection cancer center	
		Outdated information in the province’s cancer registration system	
		Limited access to screened patients’ information across different sectors	
	SIB Weaknesses	Inadequate referral system between care levels	“The SIB system works for primary care, but it does not connect with the secondary level. So, when a midwife assesses a patient and refers them to the secondary level, we cannot follow up because there’s no connection. Since we do not have any other way to follow up, we end up using social media like WhatsApp to keep track of our patients who are referred to the second level
		Lack of an online booking system	
		Lack of recording the screening results	
		Inability to register foreign patients who living in Iran in the SIB database	
		Lack of sharing screening information between providers at different levels	
		Absence of a data quality control mechanism	
		A time-consuming process to determine the level of cancer risk	
		Lack of an active queue system between health care levels	
		Delays in updating statistics in the SIB system	
		Lack of an integrated follow-up mechanism for suspected patients	
		Limited access to all necessary data in the SIB database	
Medical Facility	Medical Facility	Failure to use appropriate devices to protect patient privacy in comprehensive health centers	“In terms of medical equipment’s, we mostly go to the midwife’s room for examinations, because there is no place to examine breast.”
		Lack of replacement equipment in the early diagnosis center	

The health information system is crucial at the primary care level. The integrated health record system, known as “SIB,” is the predominant information system used for recording public health data in Iran. All health-related data within primary health services are recorded in the SIB. However, participants identified several weaknesses related to the SIB, including inaccuracies and incompleteness in the population register, time-consuming risk evaluation using the breast cancer risk assessment tool, and a lack of linkage between the SIB and other databases (governmental, private, and cancer registry). Additional issues include the absence of electronic health records at the screening level (EDC center), an inadequate referral system between different levels of care, no online booking system, failure to record screening results in the relevant database, lack of data quality control mechanisms, and insufficient information sharing or promotion of screening among providers across different levels (see Table 2).

Mammography and sonography are the only medical facilities utilized at the primary and secondary care levels. The primary challenges in medical facility category include a limited number of these facilities, which contributes to high workloads and a lack of substitutes for medical equipment in case of breakdowns. Additional issues involve inadequate maintenance of the facilities and deficiencies in user proficiency (see Table 2).

(b) Managerial barriers: Our findings indicate that the health system’s oversight of the national breast cancer screening program is inadequate. Although specialized national committees within the Ministry of Health and Medical Education (MOHME) have developed and disseminated guidelines and protocols for breast cancer screening to the provinces, there is a lack of active supervision to ensure compliance with these guidelines and assess provincial performance.

Main barriers in this dimension include the absence of a well-defined mechanism for identifying individuals eligible for screening, insufficient processes for individually inviting women to participate in cancer screening, and inadequate systems for referring screen-positive cases and reporting screen-negative results. Additionally, there is insufficient monitoring of non-responders to follow-up, a lack of an integrated education program, resource constraints, and a disconnect between primary and secondary care levels in the screening pathway. Other issues include the admission of patients from external referral systems based on self-referral without prior invitation from healthcare providers, the absence of a monitoring and evaluation mechanism for the cancer screening program’s performance, and a lack of a clear plan to enhance population participation (see Table 3).

**Table 3:** Managerial barriers.

Theme	Category	Subcategory	Quotes
Managerial barriers	Planning	Unclear organizational structure of early detection cancer centers within the health system	“The early detection center is located in a public hospital, and patients must pay for screening services at the hospital cashier. However, the hospital manager claims that the center is not part of the hospital but is instead governed by the Primary Health Care (Health Deputy). As a result, we do not receive any budget allocation from the hospital, leading to significant weaknesses in our required equipment.”
		Defects in human resource planning specific to the screening program	
		Failure to establish an integrated system at both primary and secondary care levels	
		Admission of patients from other areas (self-referrals and screenings without invitation or provider advice)	
		Lack of awareness among healthcare providers regarding national protocols and guidelines	
		Absence of an integrated education program	
		A high level of hierarchy within centers	
	Performance monitoring and evaluation	Lack of a monitoring and evaluation mechanism for CSP performance	“I had a biopsy at the doctor’s office, and it cost me 2 M. The mammography and ultrasound were also really expensive in the private sector.”
		Absence of an evaluation and accreditation system for health, particularly in screening programs	
		No specific program to monitor and report statistics as requested by higher authorities	
		Conflict of interest, such as referring patients to private sector	
	Resourcing	Lack of prioritization in funding for equipment purchases and resource allocation for physical space development	“One reason for the shortage of staff in public health is that screening services are not profitable. As a result, specialists are reluctant to work in this area due to low payment”
		Absence of performance-based incentive programs for comprehensive health centers	
		Costs not being fully covered (e.g., payment required at the secondary care level, leading to reluctance among some patients to follow up)	
		Insufficient financial incentives for specialists in early detection centers	
		Resource constraints	
	Mechanisms	No well-defined mechanism for identifying the population eligible for screening	“Midwives have to prioritize their work. So, if a pregnant woman comes in for prenatal care, she gets priority over someone coming in for another reason.”
		Insufficient mechanism for individually inviting women to cancer screening	
		Inadequate referral mechanism for screen-positive patients and reporting of screen-negative results	
		Failure to define a patient tracking system (target population, suspected cases, and confirmed cases)	
		System defects in some reports requested by higher authorities, requiring manual entry in Excel format	
		Lack of a prioritization system between screening clients and other clients (e.g., vaccinations and prenatal care, due to shared staff)	
	Community participation	Absence of a coherent public education program (relying on opportunistic and passive education methods)	“We have a Week Against Cancer where we inform the public and hold training sessions in various locations.”

(c) Service delivery barriers: At the primary healthcare level, comprehensive health care centers serve as the initial point of contact for preventive medicine. After identifying high-risk women, these centers refer them to an EDC center for further evaluation. The findings indicate that public access to cancer screening services was enhanced by the introduction of EDC centers at the secondary healthcare level. Additionally, due to cultural and religious considerations, all clinical breast examinations at the primary care level are conducted by female staff.

However, there are several barriers in health service delivery. The first category pertains to the healthcare system. Participants identified several issues adversely affecting access to the screening program, including the use of the same healthcare providers for multiple services in the public health sector (e.g., immunization and maternal care), the absence of a queue management system, overcrowding during peak times, long waiting periods for appointments, restricted availability to clinics, inadequate follow-up of test results, inaccurate clinical breast examinations performed by midwives, and the practice of referring patients to secondary care (mammography) without proper triage. Additionally, the lack of visits by primary care physicians (PCPs) before referral to secondary care and the challenge of scheduling screenings, particularly for employed women due to the centers’ morning hours, further complicate access to the screening program.

According to the women interviews’ results, barriers preventing women from participating in the cancer screening program include a lack of knowledge about screening, cultural and religious objections to mammography and clinical breast examinations, and fear and anxiety related to cancer and screening outcomes (such as cancer diagnosis, pain during the procedure, and false positive results). Other challenges involve inadequate financial coverage for tests in the private sector, inability to afford indirect costs associated with screening such as transportation, resistance to undergoing mammography due to a lack of symptoms and a perceived sense of good health, ineffective communication between doctors and patients, and mistrust of public versus private healthcare facilities. Additional barriers include lower motivation for annual screening, discomfort with disrobing for the examination, busy schedules, negligence regarding health, and insufficient family support (particularly from spouses). The findings also indicate that women have limited knowledge about risk factors, symptoms, screening frequency, and the appropriate age for breast cancer screening. Therefore, there is a need to enhance patient education regarding the importance of breast self-examination (BSE) and available screening options (see Table 4).

**Table 4:** Service delivery barriers.

Theme	Category	Subcategory	Quotes
Service Delivery Barriers	Availability	Using shared health providers for different services in the public health sector (e.g., immunization, maternal care and also screening)	“This center has only one specialist.” “Our peak time is from nine to eleven in the morning, so all services should be available during those two hours.”
		Unclear schedule for physician availability and center closure in the absence of a doctor	
		Irregular attendance of personnel at the workplace due to the doctor’s absence	
		Absence of an appointment system	
		Congestion during morning hours at comprehensive health centers	
		Difficulty in allocating time for screening, especially for employed women, due to the centers’ morning hours	
		Long waiting times for appointments	
		Limited availability of doctor visits in early diagnosis centers	
		Failure to protect patient privacy during examinations, lack of patient comfort	
		Failure to follow up patients referred from first to second and from second to third levels	
		Long distances to the early cancer diagnosis center within the province and high transportation costs, especially for patients coming from other cities	
		Lack of financial coverage for diagnosis tests in secondary care	
		Inappropriate and small waiting room space for patients	
		Limited operating hours of the early diagnosis center (one day a week)	
		Absence of a separate cashier for covering expenses at the early diagnosis center, requiring patients to rely on the public hospital cashier	
		Long and inefficient process for receiving mammography and ultrasound results	
	Patient attitude	Lack of knowledge about screening	“I think the sonography at the public center wasn’t very accurate. They identified something suspicious but did not explain it clearly. I went to the private sector for a more accurate assessment.”
		Cultural barriers to mammography and clinical breast exams	
		Fear and anxiety about cancer and screening outcomes (e.g., cancer diagnosis, pain during the procedure, false positive results)	
		Resistance to undergoing mammography due to the absence of symptoms and a perception of being healthy	
		Lower intention to participate in annual screening	
		Discomfort with removing clothing during the screening process	
		Unpleasantness of the mammography process	
		Distrust of public healthcare facilities compared to private sector options	
	Acknowledgment and acceptance of the patient	Lack of awareness among patients about symptoms, the importance of screening, and risk factors, especially within the target population	“It was a hassle trying to get an appointment for a mammogram at the public center, so we ended up going to private sector.”
		Lack of doctor involvement in patient care	
		Preference for private sector services due to perceptions of higher quality	

## Discussion

This qualitative study aims to identify the challenges associated with implementing breast cancer screening programs. Through interviews with healthcare professionals and women, the identified challenges are categorized into three primary dimensions: infrastructural, managerial, and healthcare service delivery barriers.

There is limited evidence for the efficacy of CBE as a population-based screening modality in Iran, where mammography is not routinely performed. Although mammogram machines are expensive, and their only application is in breast imaging, limiting their accessibility in only in one center in the governmental health sector in Iran. Literature suggests that CBE as part of comprehensive breast health awareness may have value in improving the opportunities for early diagnosis of a (potential) future breast cancer (20, 21).

Based on our finding, several infrastructure-related barriers affect the effectiveness of cancer screening programs. These include a shortage of personnel, insufficient number of EDC centers; inadequate facilities within health centers; and a limited availability of mammography and sonography units. Our results are supported by other studies. For instance, Prisca et al. highlight staffing shortages and inadequate on-the-job training in midwifery services for breast cancer screening as barriers to the integration of clinical breast examinations (CBEs) with cervical cancer screening (CCS) services in primary care clinics (22). Similarly, Mosquera et al. found that there is a lack of professionals adequately trained in screening protocols and guidelines (23).

Limited human resources for breast and cervical cancer screening became factor that influenced the ability of the programs to meet their targets. Then, the availability of these resources is contingent upon the education system’s capacity to produce diverse cadres of healthcare providers and the health system’s ability to attract, motivate, and retain them (24).

Offering appropriate diagnostic and treatment services involves ensuring access, which is determined by the available infrastructure and workforce. Some programs have used mobile units to improve access to screening and diagnosis. However, evidence of the effectiveness of these interventions is currently limited (25, 26).

Health Information Systems (HIS) in cancer screening program faced several critical challenges. First, the target population is not accurately registered for identification, invitation, and follow-up related to screening in the HIS. Additionally, the presence of disparate and non-integrated databases across different healthcare levels exacerbates these issues. Furthermore, there is a notable lack of data quality control mechanisms to ensure the accuracy and reliability of the information collected. These challenges significantly hinder the effective management and evaluation of cancer screening programs. The primary finding of Tarver et al. is that the positive impact of Health Information Technology (HIT) varies across the cancer continuum. Specifically, analyses targeting diagnosis and treatment were less likely to find a beneficial effect when compared to analyses targeting prevention (27). Mohammadi et al. found that the quality of cancer registry data is relatively low regarding completeness and validity (28). Mosquera reports that the population register is neither accurate nor complete, and it is not updated in a timely manner with changes in contact information (23). Nease and colleagues found that a reminder system led to increases in cancer screening in primary care practices (29). However, based on the literature HIT interventions targeted to patients were less likely to find a beneficial outcome than articles that use HIT interventions targeted to physicians. It seems that various applications of HIT can be used differently throughout the different continuum levels such as prevention, detection, diagnosis, and treatment. Despite the need for a less complex system relative to treatment facilities, there remains a significant gap in the development of an accurate and comprehensive information system for recording client data at screening centers in our country.

A major identified problem is the lack of an effective referral system and the disconnect between primary and secondary care levels within the screening pathway. Key barriers in the managerial dimension include the absence of a mechanism for identifying and inviting women eligible for cancer screening, as well as inadequate monitoring of non-responders to follow-up. Population-based screening is a pathway that begins with the identification of each individual within the target population, followed by personalized invitations and continuous follow-up throughout the entire clinical pathway. This approach ensures equitable access to screening, diagnostic, and treatment procedures. However, when the healthcare system is fragmented, hindering patients from navigating the full continuum of care, diagnostic delays are likely to occur. To improve the referral system, healthcare settings should adopt a more comprehensive approach that integrates multiple elements such as clinician education, organizational culture change, continuous quality improvement, and coordination between public health and therapeutic care levels.

Overcrowding during peak times, long waiting periods, inaccurate triage, and lack of general practitioners (GPs) at the primary level of public healthcare were the identified barriers in service delivery in cancer screening. The delivery of effective diagnostic and treatment services relies on robust program management and the establishment of comprehensive networks of providers across the community. The provision of appropriate and timely clinical preventive services is essential to reduce the morbidity and mortality associated with breast cancer. Effective cancer screening programs should accurately identify women eligible for breast cancer screening and ensure access to comprehensive diagnostic and treatment services within a reasonable timeframe. While lack of general practitioners (GPs) at the primary level of public was a critical factor in inaccurate triage. Richardson et al. emphasized that women with an abnormal screening test should undergo a comprehensive diagnostic evaluation within 60 days, followed by the initiation of treatment within 60 days after diagnosis. This timeline is particularly important because uninsured women, ethnic minorities, and those with lower socioeconomic status are at a greater risk of delays and incomplete follow-up after an abnormal screening result (30). Some participants reported experiencing more than two-month delay in the diagnostic process due to a lack of available physicians in the EDC center which can be associated with a more advanced stage of disease at diagnosis and poorer survival (31, 32). Simultaneously, educating primary care providers to identify the early signs and symptoms of breast cancer is essential for timely referrals within the healthcare system.

The interviews with women revealed several barriers to breast cancer screening, primarily related to their knowledge and attitudes. These barriers include insufficient awareness, low perceived risk, fear and anxiety regarding cancer, inadequate family support, and cultural and religious considerations to examinations. Muslim women often hold socio-ethical, cultural, and religious misconceptions about health, practices, and the nature and causes of breast cancer. Cultural barriers and religious values have been shown to influence their health behaviors, such as maintaining modesty when selecting health interventions (33). Based on a review, the most common barriers to breast cancer screening across populations were race/ethnicity, low socioeconomic status, and educational levels, as well as lack of family history of cancer and being single (34). Some individual-level barriers, such as limited reach of the program, and low health literacy, were consistent with findings from other studies (7, 35, 36). To increase patient demand for cancer screening, it is essential to expand health education initiatives. Previous studies have indicated that nearly 60% of women did not know how to perform breast self-examinations (BSE) (37–39). Allaire et al. emphasized patient navigation as an essential technique for delivering timely and high-quality cancer screening to medically underserved women. This strategy is expected to enhance both life-years and overall health outcomes (30).

Popalis et al. report that educational sessions with community health workers or one-on-one patient interactions can improve cancer screening (40). Other research recommends several interventions that consistently enhance participation in cancer screening, including patient navigation strategies, pre-screening reminders (41), general practitioner endorsement, educational outreach, peer counseling, and small media initiatives (42).

### Strengths of the study

One of the key strengths of this study is the use of triangulation, which enhances the reliability and validity of the findings. By employing a combination of interviews, direct observations, and document analysis, the study integrates multiple perspectives and sources of data, allowing for a more comprehensive understanding of the breast cancer screening workflow. In the qualitative phase, we interviewed with two groups—women undergoing screening and healthcare professionals involved in the process—provide valuable insights from both the patient and provider perspectives. This approach not only helps to validate the findings through cross-referencing but also captures a holistic view of the screening process, including potential challenges, patient experiences, and professional practices.

### Limitation

This study encountered a few limitations; firstly, we used a convenience sample in the women group and they were selected from the comprehensive health service and early detection cancer units. Although we continued interviews until saturation, it is unclear that our participants are representative of other patient populations. Meanwhile, we did not categorize the women-based demographics, health literacy, or other factors that might have an effect on their perspectives. Secondly, by design, qualitative research has limited generalizability, although because of lack of data, we have not any choices to catch the challenges in cancer screening program.

## Conclusion

This study identifies key barriers to implementing breast cancer screening programs in Iran, including infrastructural deficiencies, managerial inefficiencies, and service delivery challenges. Inadequate facilities and HRH, ineffective referral systems significantly hinder the CSP effectiveness. To improve the referral system, healthcare settings should adopt a more comprehensive approach that integrates multiple elements such as clinician education, organizational culture change, continuous quality improvement, and coordination between departments. Additionally, barriers related to knowledge, perceived risk, and cultural factors among women further complicate screening efforts. To overcome barriers to breast cancer screening among women, strategies should focus on increasing awareness through educational campaigns and improving the accessibility of screening services. Overcoming these barriers is crucial to improving the accessibility, efficiency, and overall effectiveness of breast cancer screening programs, ultimately enhancing patient outcomes. It needs to emphasize, performing a situational analysis in each country in terms of the political, economic, and social context; infrastructure (equipment, facilities, HIT), HRH capacity, financial resources, healthcare system capacity, public awareness, legal and regulatory frameworks, and cultural factors is necessary before introducing any new intervention.

## Data Availability

The raw data supporting the conclusions of this article will be made available by the authors, without undue reservation.
