# Supplementary material for: Implementation and performance barriers in Iran’s breast cancer screening program: a qualitative case study
Source: Front Public Health. 2025 May 9;13:1490191. doi: 10.3389/fpubh.2025.1490191 (PMC12098275; doi:10.3389/fpubh.2025.1490191)

**Supplementary 1: Document Analysis Summary of National Breast Cancer Guidelines**

| **Document Analysis Summary of National Breast Cancer Guidelines** | | | | | |
| --- | --- | --- | --- | --- | --- |
| **Document Title** | **Publication Date** | **Recommended Age for Screening** | **Frequency of Screenings** | **Types of Tests** | **Roles And Responsibilities** |
| Breast cancer screening and prevention guidelines (for doctors and midwives) | - | Women aged 20-69 | Annual | 1. breast self-examination  2. Mammography | 1. Midwife/ GP: breast self-examination  2. Radiologist /Specialist/ Surgeon: Mammography/ Biopsy |
| Essential Interventions for Noncommunicable Diseases in the Iranian Primary Health Care System "IRAPEN"  (Midwife) | 2017 | Women aged 30-69 | Annual/Biennial | 1. breast self-examination  2. Mammography | 1. Midwife/ GP: breast self-examination  2. Radiologist /Specialist/ Surgeon: Mammography/ Biopsy |
| Essential Interventions for Non-Communicable Diseases in the Iranian Primary Health Care System "IRAPEN"  (Behvarz/Health Care Executive Manual) | 2017 | Women aged 30-69 | Annual/Biennial | 1. breast self-examination  2. Mammography | 1. Behvarz/Health Care Executive Manual: breast self-examination  2. Radiologist /Specialist/ Surgeon: Mammography/ Biopsy |
| National Guidelines for Early Detection of Cancer in IRAN | 2021 | Women aged 30-69 | Annual/Biennial | 1. breast self-examination  2. Mammography  3. sonography | 1. Midwife/ Behvarz/Health Care Executive Manual /GP: breast self-examination  2. Radiologist /Specialist/ Surgeon: Mammography/ Biopsy  3. Specialist/ Surgeon: sonography |

**Supplementary 2: Interview Questions**

Health care provider questions

**Infrastructural barriers:**

1. How do you evaluate the availability of skilled and trained personnel required for performing breast cancer screening?
2. How do you evaluate the level of job satisfaction and motivation among the personnel involved in breast cancer screening?
3. How do you evaluate the physical infrastructure of the service facility in terms of providing care to patients and offering rest areas for staff?
4. In your opinion, does the quantity and quality of the workforce involved in breast cancer screening meet the needs of the community?
5. What challenges exist in this area?
6. **I**s there an appropriate information system (such as a database, website, etc.) for monitoring the incidence, prevalence, and mortality trends of cancer? If yes, please specify the system and evaluate its data quality, ease of access, user-friendliness, and overall usability.
7. Does the information technology system (information system) provide adequate support for recording cancer data? If yes, please specify the system and evaluate it in terms of user-friendliness, ease of access, ease of reporting, data quality, and other relevant factors.
8. Has specialized personnel (such as epidemiologists) been involved in the analysis of data from the information systems?
9. Can you explain the accuracy and quality of data recording?
10. Does the existing information technology system support monitor the incidence of cancer and mortality trends?
11. **I**s there a shortage of essential medications, equipment, and supplies for screening?
12. Are modern technologies being used for breast cancer diagnosis?

**Managerial barriers:**

1. Are there any policies and guidelines in place for the prevention and control of breast cancer?
2. Is there a national guideline or protocol regarding the procedures for breast cancer screening? If yes, what is your opinion on the relevance and up-to-datedness of this guideline or protocol in relation to current conditions?
3. Have any measures been taken to educate and raise awareness among the eligible population to encourage regular screening? (Such measures might include workshops, seminars, advertising campaigns, etc.)
4. Are there specific processes and outcome indicators in place for monitoring and evaluating the screening program?
5. Have you encountered financial challenges in promoting and advocating for the screening program (e.g., through workshops, campaigns, etc.), employing adequately skilled personnel and providing their training, accessing necessary equipment and medications for patients, or offering services to patients? If yes, please evaluate the financial resources required for each of these specialized activities.
6. Does the current screening system have the required effectiveness? If not, what are your suggestions for improving or changing it?

**Service delivery barriers (health care providers):**

1. Are screening services provided to patients in a timely manner (without long waiting times)?
2. Do you think access to screening services is different for people living in rural areas or towns compared to those living in cities? What are the problems you think exist in this regard?

Women questions

1. Who is considered a high-risk individual for breast cancer?
2. What factors increase or decrease a woman's risk of breast cancer? Please share any information you know or have encountered.
3. What have you heard about the recommendations for how frequently women should undergo mammograms (or clinical breast examinations) based on their breast cancer risk?
4. What are the various reasons women choose to undergo mammograms (or clinical breast examinations) or decide not to get them?
5. what are your thoughts on the idea of getting a mammogram or clinical breast examination every year, or every two or three years, depending on your risk?
6. What would have the greatest impact on your decision to follow or not follow your physician’s recommendations?
7. What have been your previous experiences with mammography (or clinical breast examination)? Please identify any barriers and facilitators (e.g., access, cost, communication with health professionals, physical factors such as waiting room conditions, accommodation, etc.).

**Supplementary 3: Conceptual framework**


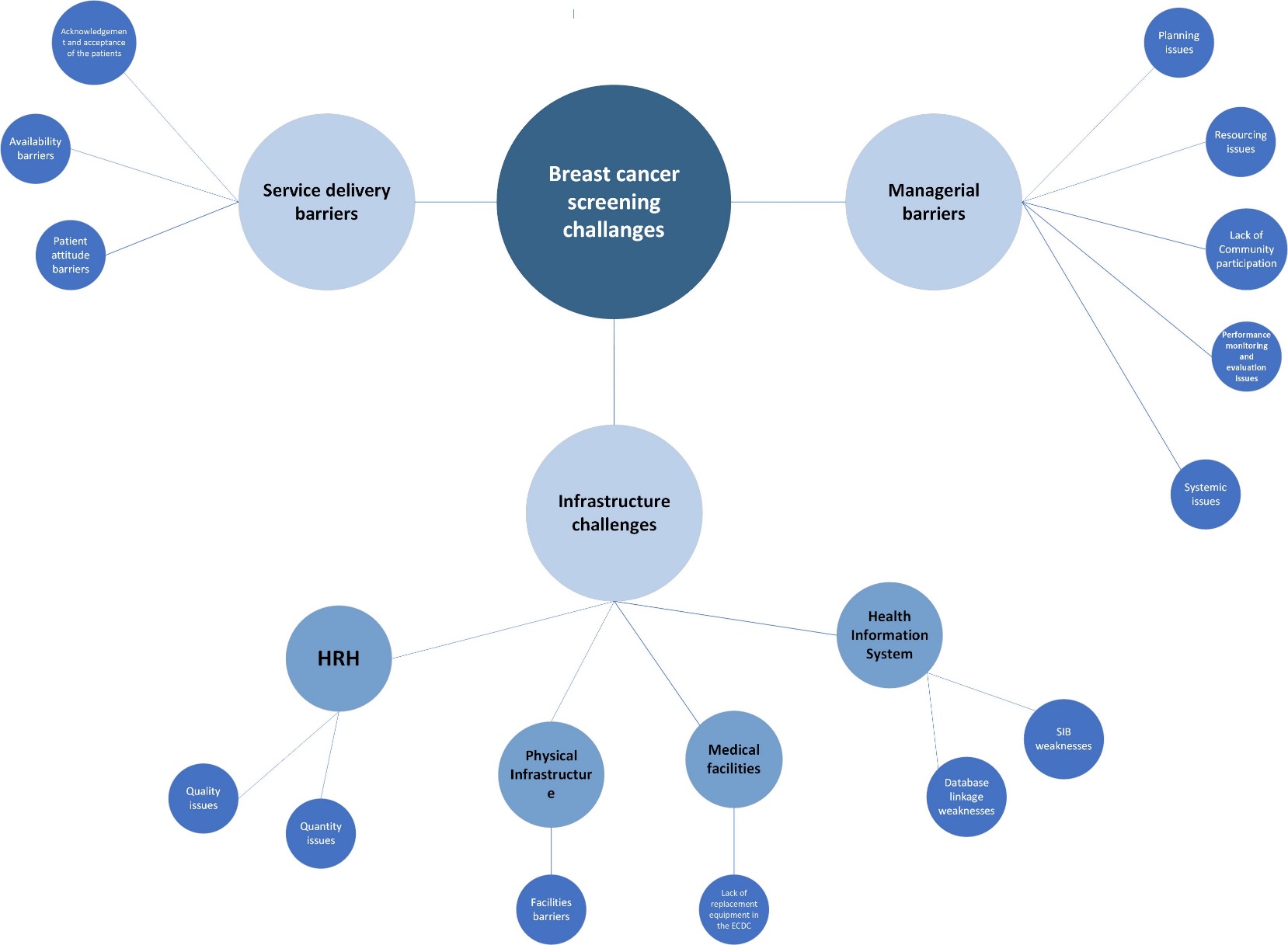

Supplement: Supplementary file 1 [file Data_Sheet_1.docx]
